# Ten simple rules to make your research more sustainable

**DOI:** 10.1371/journal.pcbi.1008148

**Published:** 2020-09-24

**Authors:** Anne-Laure Ligozat, Aurélie Névéol, Bénédicte Daly, Emmanuelle Frenoux

**Affiliations:** 1 Université Paris-Saclay, CNRS, LIMSI, Orsay, France; 2 ENSIIE, Evry-Courcouronnes, France; Carnegie Mellon University, UNITED STATES

## Introduction

Sustainable development can be defined as a principle that regulates human activity without causing irreparable damage to the Earth's natural system. It also aims to preserve resources so that future generations can benefit from them as much as present generations. To address the global challenges today's world faces to manage the impact of human activity on the environment, the United Nations have defined a set of sustainable development goals to be achieved in the next decade [1].

The climate changes induced by human activities have been accelerating alarmingly as reported by scientists since 1979 [2]. Scientists can observe an increase in pollution (e.g., depletion of oxygen in water, eutrophication), natural resource scarcity, and a significant and accelerated loss of biodiversity. All these changes have led geologists to propose the Anthropocene as a new geological epoch, reflecting the impact of human activities on Earth's ecosystems[3].

To mitigate this effect, a paradigmatic shift represented by sustainable development is needed in all fields of human activities, including research. As individuals and researchers, we are concerned with these challenges and deeply aware of the necessity to be more sustainable. But in practice, what does this entail? How can a researcher's activity be “sustainable,” and how do we integrate sustainable practices into research projects? Where do we start?

There is currently no global policy from French research institutes to federate collective action of the scientific community towards sustainable development goals, but working groups focusing on sustainable development have published recommendations [4]. There are also examples of good practice in the United Kingdom (S-Labs [5], Laboratory Efficiency Assessment Framework [6]) and the United States (International Institute for Sustainable Laboratories [7], My Green Lab [8]).

Taking action to address the emergency situation is not only a moral responsibility we have as citizens but also a necessary contribution to gathering an understanding of the impact of research activities on the environment and how to make them more sustainable.

This article is the result of the work carried out by the "sustainable development" committee created at the French Laboratoire d'informatique pour la Mécanique et les Sciences de l'Ingénieur (LIMSI) [9] in 2019 to bring together researchers interested in addressing these questions in the context of the activities of our laboratory, which conducts theoretical and experimental research in a diversity of scientific fields, including fluid mechanics, energetics, human language technology, human machine interaction, medical informatics, and augmented and virtual reality.

The first task of the committee was to assess the carbon footprint of research activities over the year 2018 [10]. The next task is to analyze the results of this study and draw a roadmap towards reducing the carbon footprint and, more generally, the environmental impact [11] of our research activities in subsequent years.

Herein, we propose a list of actionable rules to facilitate the contribution of anyone in the community towards sustainable research. We selected a set of rules that address a variety of topics with different levels of potential impact with the goal of illustrating the breadth of possible actions.

## Rule 1: Cherry-picking is allowed

### Why does it matter?

It can be daunting to think about all you should be doing to strive towards sustainability. However, in the words of popular wisdom, Rome wasn't built in a day, and every little thing helps. We want to acknowledge here that integrating sustainability into our research is a big step for many of us and that this change may need to be gradual, according to behavioral theory [12]

### How to address it?

You can start your path towards sustainable research today by picking only one of the suggestions below and committing to it. Depending on your particular field of research or interests, some rules may be easier to implement than others.

## Rule 2: Be informed

### Why does it matter?

Information is the foundation of sustainable action. According to the Paris Agreement [13], we have a global goal of achieving carbon neutrality in 2050 and halving current carbon emissions by 2030. Drastically reducing the carbon footprint of human activities can only be achieved if we are well aware of the specific impact of the different carbon-emitting activities.

### How to address it?

Research institutes should ensure that their staff receive some **training on environmental issues** related to energy, climate and biodiversity. Training courses are available on this subject, including Massive Open Online Courses (MOOC) through popular platform such as Université Virtuelle Environnement et Développement Durable (UVED) [14] in French and Coursera [15] in English. The sustainability literacy test [16] is a tool approved by the United Nations for learning general knowledge relating to the environment that could also be used to enhance workers’ and students’ knowledge. In addition, general public documents such as summaries of Intergovernmental Panel on Climate Change (IPCC) reports or reports from think tanks such as the The Shift Project or the Green Alliance can also be used as information material, as well as documents from environmental nongovernmental organizations such as 350.org or Greenpeace. Awareness of these issues will facilitate their inclusion in lab operations and scientific work.

Evaluating the **carbon footprint** of your lab/institute is an excellent start. An information search for reports of carbon footprint assessments conducted by laboratories or institutes in the same field can also help identify major trends. Typically, for research labs, travel accounts for a significant portion of carbon emissions. Other major sources of carbon emissions include electricity used for building operations as well as computer power. In France, the labos1point5 [17] collective is offering support to labs interested in assessing their carbon footprint.

Carbon footprint assessment can also be done at the scale of specific research activities. Researchers can apply their knowledge of how to evaluate and compare the impacts of two alternatives. For example, from an environmental point of view, is it better to continue using legacy equipment that may require more power or to invest in new equipment that will require less power but incur environmental construction costs [18]? Which videoconference system incurs the lowest energy consumption [19]?

Although these questions may be hard to answer, some of them can be addressed using widely recognized methodologies, such as Life Cycle Assessment (LCA). LCA enables the assessment of environmental impacts of a service or product by taking into account all the stages of its life cycle according to different criteria, including but not limited to carbon dioxide CO_2_ measurement. This again requires that research staff be trained on these methodologies, in order to apply them properly. The results obtained may differ substantially on a case-by-case basis because the assessment is dependent on the location and specific set-up. For example, whether the electricity used comes from low-carbon sources will have an impact. In some cases, the LCA will nevertheless remain difficult if key relevant data (e.g., electric power provenance) is not available.

## Rule 3: Prefer train over plane

### Why does it matter?

The main source of global CO_2_ equivalent emissions in several research institutes is travel. For example, at LIMSI, in 2018, travel accounted for 50% of the total emissions [10], including about 35% for transportation to attend scientific meetings or conduct field work and an additional 15% for employees’ commutes. Other case studies at two academic institutions in Switzerland and in the US also found travel to account for a large share of their carbon footprint, with air travel alone accounting for 30% of all CO_2_ emissions [20, 21].

**Fig 1:**
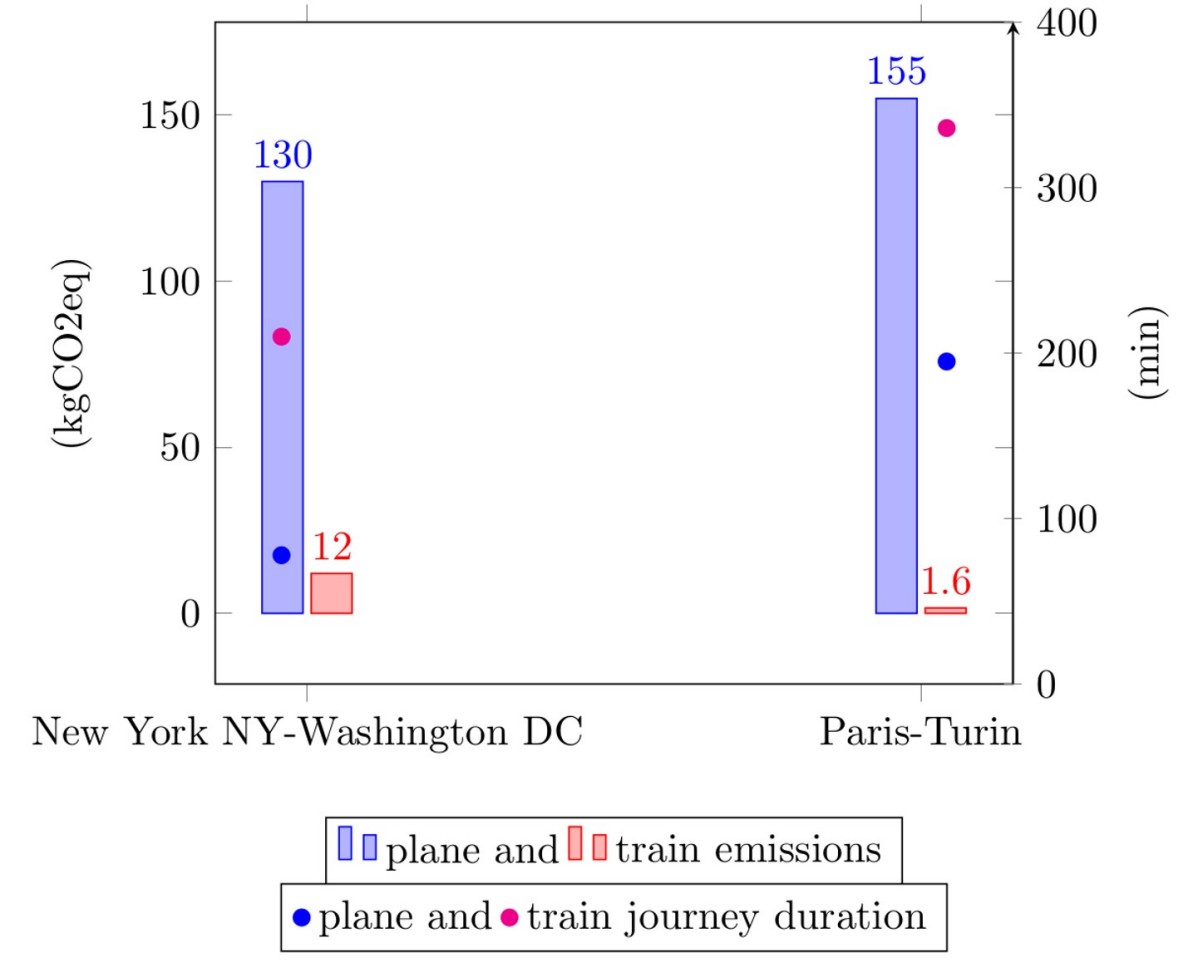
Comparison between plane and train travel in terms of time and CO_2_ emissions (kg) for two sample itineraries. Plane emissions were calculated with the https://co2.myclimate.org/en/flight_calculators/newmyclimate flight calculator. Flight durations are estimated using https://www.expedia.fr/expedia. Train emissions and durations come from oui.sncf/SNCF for the Paris–Turin journey and from https://www.virail.frvirail for the New York, New York–Washington, DC journey.

Fig 1 presents a sample comparison between plane and train travel for two sample itineraries to illustrate the CO_2_ emission gain offered by train travel for medium range journeys: one international journey within Europe (Paris–Turin, 584 kilometers/363 miles) and one domestic journey withing the US (New York, New York–Washington, DC, 474 kilometers/295 miles).

This data shows that carbon emissions associated with train travel represent a mere fraction of those associated with plane travel (9.2% for New York/Washington and 1% for Paris/Turin), while travel duration increases by about 2 hours (precisely 2 hours 12 minutes for New York/Washington and 2 hours 21 minutes for Paris/Turin), which is arguably equivalent to the time associated with air travel formalities, including city/airport commute and airport security procedures.

### How to address it?

This data shows that traveling by train instead of plane can massively reduce the footprint of academic travel. While travel is perceived as essential to a researcher's activity [22], it was also shown that air travel has a limited influence on academic professional success [23] for senior researchers. Therefore, train travel should be favored whenever possible. We also encourage researchers to reduce their travel footprint by favoring attendance to scientific meetings in locations that can be reached by train and to limit their conference travel, which scientists seem willing to do [24, 25].

This rule applies mainly for short distance travel, which only accounts for a fraction of academic travel. Typically, the train is not an option for traveling from Paris (France) to San Franciso, California (US). The impact of a Paris–San Francisco round-trip flight in terms of CO_2_ emissions (2.9 tons according to myclimate) is roughly equivalent to 10 times that of a domestic Paris–Toulouse round trip (314 kg according to myclimate). Favoring train over plane will not reduce emissions related to long-distance trips and thereby may have limited impact over global travel-related emissions if long-distance travel accounts for the majority of travel. As a result, it is necessary to limit long-distance travel by assessing the need for travel, encouraging local collaboration, and adopting publication methods that restrict travel, such as journal publications or domestic conferences.

## Rule 4: Take advantage of remote participation

### Why does it matter?

As discussed above, it is necessary to limit long distance travels because they incur a high level of carbon emissions within the overall travel category, which is a major source of emissions for research institutions.

### How to address it?

Remote participation in conferences and webinars can be encouraged and facilitated.

Pioneer events showed that entirely remote conferences can be organized to the satisfaction of an overwhelming majority of participants: 10 years ago, computational biologists published a set of guidelines for organizing such events as an effective low-cost educational strategy [26]. More recently, the University of California at Santa Barbara also devised a plan to organize remote conferences [21] in which conference participants were invited to record their talks ahead of the event; the videos were then made available on the conference website, and direct interaction with the authors was supported by message forum within the conference timeline. A similar set-up was used for the Cochrane Colloquium 2019 after the event had to be cancelled due to local political events [27]. A more complete list of conferences aiming to limit their carbon emissions is available at https://www.appropedia.org/List_of_low-carbon_conferences, and recent guidelines provide support for organizing non–real-time events [28]. The recent global public health crisis due to the COVID-19 outbreak is providing additional motivation for organizing remote events[29].

These events show that it is technically feasible to engineer fully remote participation in conferences. However, one of the benefits of conference participation is the networking and personal interactions between colleagues occurring during coffee breaks and social events. For this reason, the scientific community may want to continue fostering in-person meetings. To support this middle-ground solution, remote participation can be promoted as a systematically available option for all conferences and meetings [30]. This way, researchers may balance their conference attendance between in-person and remote attendance.

Another option that can help reduce the carbon footprint of conferences is to create small groups of participants that can meet in person in a local venue to attend a remote event. The 2010 "Signs of Change" conference was organized according to this model and offered five locations throughout New Zealand for participants to meet [31].

## Rule 5: Work collectively and reproducibly

### Why does it matter?

Many of the resources allocated to research activities—including resources that have an impact on the environment—are wasted due to a multiplicity of factors related to the organization, planning, and evaluation of research [32].

For example, the inadequate use of statistical methods or the fact that novelty is valued more than reproducibility results in waste that could be otherwise avoided. The practice of sharing protocols, research material such as code, and results contributes to collective work and avoids waste in the form of duplicate efforts.

### How to address it?

Applying the classic scientific method is particularly useful here. First, do a thorough literature search to identify useful related work to a new project. If existing systems or models addressing the task at hand are identified, they should be reused. Novelty and originality does not necessarily come from new methods; an existing method can be novel if applied to a new context or used differently from usual practice. Furthermore, reusing existing material can also bring added value by demonstrating its reproducibility, documenting a reuse case from the perspective of users with a different background or experimental set-up [33, 34].

If a thorough literature search does not uncover readily usable solutions, it makes sense to develop new methods or tools. In this case, working reproducibly will help researchers make the most of their work, by documenting protocols and sharing material. In fact, the value of reproducibility is increasingly promoted by "reproducibility challenges" that seek to reproduce prominent work and gather information about the process. The events Neural Information Processing Systems (NeurIPS) 2019 Reproducibility challenge [35] and the Shared Task on the Reproduction of Research Results in Science and Technology of Language,"REPROLANG 2020" [36] are examples of reproducibility tasks in the fields of Natural Language Processing and Machine Learning. In 2020, the Empirical Methods in Natural Language Processing (EMNLP) conference added reproducibility criteria during the submission process about the description of experimental results, parameters, and datasets, based on [37] and [38], which is also a good way to promote reproducibility of research work.

## Rule 6: Encourage bottom-up sustainable initiatives

### Why does it matter?

Engaging the community on the topic of sustainability will create interest in the topic and in how it is addressed within the community. Empowering members of the community will contribute to the emergence of solutions that are tailored to the community culture and that will find stronger support within the community [39].

### How to address it?

Researchers are creative. Let them implement ideas towards more sustainable practice.

Typically, many of the initiatives cited in this manuscript came from researchers themselves and have been widely adopted. Methods for organizing remote conferences are one example which has shown increased popularity with the recent pandemic.

Supporting this type of initiative (environmental impact of research operation/policy) as a research question will help with researching and adopting some of these ideas. There are many ways this support can translate into actionable policies for a variety of actors in higher education and research: by funding researchers' work in this area, even if it is outside of their main expertise, e.g., labs could fund researchers experiments towards addressing these questions, journals could waive publication fees for this type of work, and institutes could recognize this type of contribution in staff evaluation or provide special allowance to allocate a portion of their time working on these issues.

## Rule 7: Evaluate the impact of your research practices

### Why does it matter?

Like all human activities, research has an environmental impact that we need to be aware of (see Rule 2). Until now, we have mainly discussed the impact of the research environment rather than research activities in and of themselves. The raising awareness for environmental issues combined with the increasing energy needed for implementing modern machine learning algorithms has brought about the emerging field of so-called "green" artificial intelligence, which seeks to reconcile powerful computing with environment friendly research [40].

### How to address it?

When conducting research, factor in the direct, indirect, and structural environmental impacts of your research. Direct impact covers the carbon footprint of the operational conduct of the research. For example, it includes the carbon footprint of overall research practice and can be taken into account by addressing questions such as the following: When two methods are otherwise equivalent in performance, which one has the smaller carbon footprint? Did you take computational cost and its environmental impact in your method evaluation/reporting [38, 41]?

Indirect and structural impact covers the consequences of the new knowledge or findings obtained as a result of the research. For example, improving the energy efficiency of a learning algorithm could lead to increased experimentation, which, in the long run, does not reduce energy use (this typical rebound effect is described in [18]). Indirect and structural impact can be taken into account by addressing questions such as the following: What are the consequences of the discoveries we make? Do they contribute to a more sustainable world or, on the contrary, to a runaway machine, even indirectly?

## Rule 8: Ask sustainability research questions

### Why does it matter?

Rule 2 and others have highlighted how information is key to address sustainability. Research aims to create new knowledge and therefore can have a contribution to our information on sustainability issues and how to address them.

### How to address it?

Computer science (CS) is well positioned for offering analysis of problems using predictive models, simulation and visualization methods that can be applied to a large range of sustainability problems. The National Research Council Committee on Computing Research for Environmental and Societal Sustainability has suggested that "[s]marter energy grids, sustainable agriculture, and resilient infrastructure provide three concrete and important examples of the potential role of IT innovation and CS research in sustainability." [42]

However, the benefit of work on sustainability research questions must be balanced with the impact of such research (see Rule 7). For example, although information and communication technologies are often considered as a means for reducing energy demands and emissions, a recent study [43] showed that digitalization actually increases energy consumption. Standard methodologies can be used here, such as attributional LCA (see Rule 2), which takes into account the direct environmental impacts. The more general and long-term impact of research work can be difficult to anticipate or predict. Typically, researchers investigating electricity in the 17th and 18th century may not have foreseen the climate crisis we are facing today due to CO_2_ emissions generated by electricity-powered technology. In order to assess research impact at a global level, consequential LCA can also be used. It goes beyond the direct impacts and may require multidisciplinary work involving economists, sociologists, philosophers, etc.

Furthermore, researchers can be encouraged to think about research questions outside of their main expertise (see Rule 6). For example, several French groups with an interest in advancing sustainability policies [17] drafted incentive and coercive plans to reduce airplane travel in research labs. The incentive measure consists in making it mandatory to compensate the carbon emissions linked to travel by funding compensating projects. The coercive measure consists in banning travel as soon as the yearly carbon quota defined either per lab or per researcher has been reached. They are suggesting enforcing these policies in pilot institutes as a scientific experiment to test adherence and impact.

## Rule 9: Transfer ecofriendly gestures from home to the lab

### Why does it matter?

Studies show that individual actions towards sustainability in the form of ecofriendly gestures can contribute towards achieving up to 45% of the carbon footprint reduction that needs to be achieved collectively by 2050 [44].

### How to address it?

Ecofriendly gestures practiced at home by many are also relevant in a professional setting and can have a significant impact. These ecofriendly gestures are not necessarily trivial to implement in the workplace because individuals are not directly involved in the global administration of the infrastructure. Typically, for security and logistical reasons, electric facilities cannot be openly accessible to all. Nonetheless, based on recommendations for carbon reduction [44] and our own example of carbon emissions at LIMSI [10], the following points can be brought to the attention of infrastructure management officials within an institute:

- Limit the use of (plastic) disposable material [45].
- Practice printing sobriety: think before you print and collect printed documents.
- Encourage employees to use soft modes of transport for local commutes (see also Rules 3 and 4).
- Favor local, seasonal, and vegetarian food when organizing events.
- Practice digital sobriety: use less material, make it last as long as possible, and consider donations when research use of otherwise-functioning equipment is no longer appropriate.
- Organize trash collection to encourage or facilitate recycling.
- Consider the feasibility of switching off lights and servers during off–peak-use periods.

## Rule 10: Raise awareness

### Why does it matter?

Sustainable actions have an impact both at the individual and collective level. The strength of the impact directly depends on the support of many individuals. As a result, it is important to raise awareness to convince and gain the support of others.

### How to address it?

**Fig 2:**
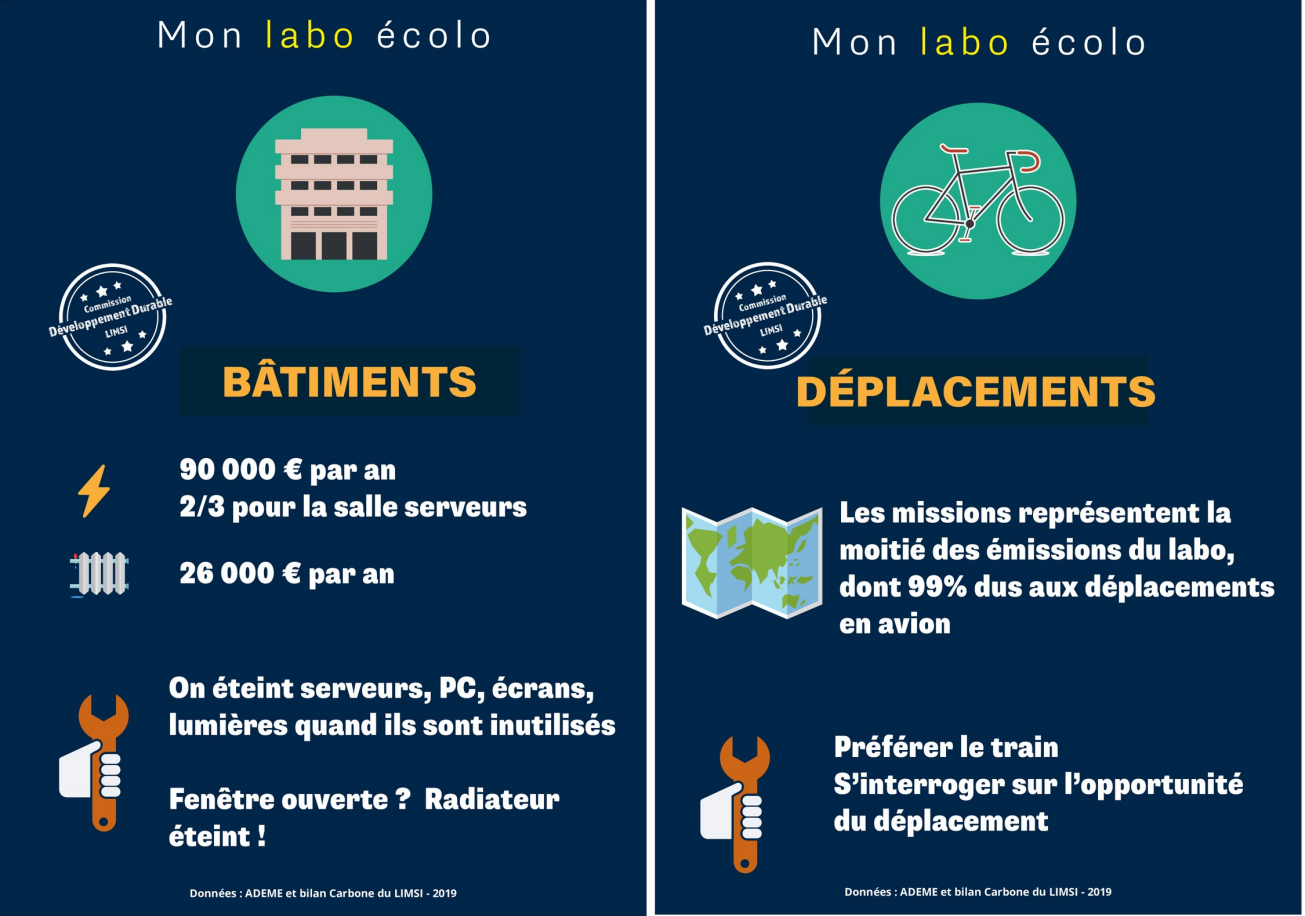
Sample handout providing information on building related power usage in Fig 2A and on travel in Fig 2B. **(A)** Translation of hand-out content into English: “My Green Lab—BUILDINGS—(electricity) 90,000 € pa, 2/3 for server room (heat) 26,000 € pa (action) Switch off servers, computers, screens, lights when not in use. Open window? Radiator off!” **(B)** Translation of hand-out content into English: “My Green Lab—TRAVEL—(map) travel is responsible for half of the lab’s carbon footprint, with plane travel accounting for 99% of the travel footprint (action) prefer train over plane; question the necessity of travel.” *Text and design by Bénédicte Daly*.

Communication officers at research institutes are in charge of internal communication using diverse means including custom visual tools and social media. They are excellent assets to support an awareness campaign on sustainability issues. For example, the contribution of LIMSI's communication director to the sustainable development committee includes the creation of a series of handouts around the theme of "Mon labo écolo" (My green lab). Fig 2 presents sample handouts produced by the LIMSI communication officer to raise awareness of lab members on major issues such as power use (Fig 2A) and travel (Fig 2B).

These documents are used at LIMSI to promote sustainable action around the lab and can be used/customized as desired.

## Conclusion

The goal of this article is to provide researchers with guidance for integrating sustainable practices into their activities. These 10 rules are positioned in the paradigm of science as it is conducted today. We posit that modern environmental and public health issues suggest the need for a massive paradigm shift. Calls to this effect have already been issued within the scientific community [46], such as the "slow science manifesto" [47].

In this situation, we stress the importance of cherry-picking and easing into change step by step to avoid being overwhelmed by the magnitude of the task.

A major step towards achieving sustainable research requires being informed about the impact of our activities as well as the impact of the simple choices we can make, as outlined by this set of 10 rules.
